# Supplementary material for: Urinary tract infections, risk factors and antimicrobial resistance patterns in heart failure patients on sodium-glucose transporter 2 inhibitors: Evidence from Jakaya Kikwete Cardiac Institute in Tanzania
Source: PLoS One. 2026 Mar 26;21(3):e0344986. doi: 10.1371/journal.pone.0344986 (PMC13020780; doi:10.1371/journal.pone.0344986)

PLOS ONE

Urinary Tract Infections and Drug Resistance Patterns in Heart Failure Patients on Sodium-Glucose Transporter 2 Inhibitors: Evidence from Jakaya Kikwete Cardiac Institute in Tanzania

--Manuscript Draft--

**Manuscript Number:**	PONE-D-25-48414
**Article Type:**	Research Article
**Full Title:**	Urinary Tract Infections and Drug Resistance Patterns in Heart Failure Patients on Sodium-Glucose Transporter 2 Inhibitors: Evidence from Jakaya Kikwete Cardiac Institute in Tanzania
**Short Title:**	UTIs and Resistance in Heart Failure patients on SGLT2 Inhibitors
**Corresponding Author:**	Ritah F Mutagonda, Ph.D<br>Muhimbili University of Health and Allied Sciences School of Pharmacy<br>Dar-es-salaam, TANZANIA, UNITED REPUBLIC OF
**Keywords:**	SGLT2 inhibitors, Heart failure, Urinary tract infections, Uropathogens, Antimicrobial resistance, Antibiotic susceptibility
**Abstract:**	Background: Sodium-glucose co-transporter 2 (SGLT2) inhibitors such as dapagliflozin and empagliflozin are increasingly used in heart failure (HF) management due to their cardiovascular benefits. However, glycosuria induced by SGLT2 inhibition may increase the risk of urinary tract infections (UTIs). Limited data exist on UTI burden among HF patients on SGLT2 inhibitors in low-resource settings.<br>Objective: This study aimed to determine the prevalence of UTIs, associated factors, and antimicrobial susceptibility patterns of uropathogens in HF patients receiving SGLT2 inhibitors at the Jakaya Kikwete Cardiac Institute (JKCI) in Tanzania.<br>Methods: A hospital-based cross-sectional study was conducted from March to June 2024 among HF patients aged ≥18 years on SGLT2 inhibitors. Data were collected using structured questionnaires and medical records. Midstream urine samples were cultured and subjected to antimicrobial susceptibility testing using the Kirby-Bauer disk diffusion method per CLSI 2024 guidelines. Descriptive statistics and modified Poisson regression were used for analysis.<br>Results: Out of 138 participants among those cultured, the prevalence of UTI was 15.9%. The most common uropathogen was Escherichia coli (50%), followed by Klebsiella pneumoniae (13.6%), Pseudomonas aeruginosa (9.1%), and Candida spp. (9.1%). Significant risk factors for UTI included age >60 years (aPR 3.77; 95% CI: 1.42–10.01), female sex (aPR 2.92; 95% CI: 1.19–7.15), and SGLT2 inhibitor use ≥4 months (aPR 3.19; 95% CI: 1.70–5.96). High resistance was observed in bacterial isolates against ampicillin (100%), tetracycline (69.2%), and ceftazidime (53.8%), whereas high susceptibility was noted against nitrofurantoin (84.6%) and meropenem (100%).<br>Conclusion: UTIs are common among HF patients on SGLT2 inhibitors, with E. coli as the predominant pathogen and a concerning resistance to commonly used antibiotics. These findings underscore the need for routine urine culture and sensitivity testing to guide appropriate therapy and promote antimicrobial stewardship, particularly in resource-constrained settings.
**Order of Authors:**	Julieth Kaaya, MPharm
	Martine Manguzu
	Deus Buma, Ph.D
	Hamu Mlyuka
	Samwel Rweyemamu, MD
	Albert Ntukula
	Helfride Ilomo, MSc
	Elias Bukundi, MSc

Powered by Editorial Manager® and ProduXion Manager® from Aries Systems Corporation

	Raphael Sangeda, Ph.D<br>Ritah F Mutagonda, Ph.D
**Additional Information:**	
**Question**	**Response**
**Financial Disclosure**<br><br>Enter a financial disclosure statement that describes the sources of funding for the work included in this submission. Review the submission guidelines for detailed requirements. View published research articles from *PLOS ONE* for specific examples.<br><br>This statement is required for submission and **will appear in the published article** if the submission is accepted. Please make sure it is accurate.<br><br>**Funded studies**<br>Enter a statement with the following details:<br>• Initials of the authors who received each award<br>• Grant numbers awarded to each author<br>• The full name of each funder<br>• URL of each funder website<br>• Did the sponsors or funders play any role in the study design, data collection and analysis, decision to publish, or preparation of the manuscript?<br><br>Did you receive funding for this work?	The author(s) received no specific funding for this work.
**Competing Interests**<br><br>Use the instructions below to enter a competing interest statement for this submission. On behalf of all authors, disclose any competing interests that could be perceived to bias this work—acknowledging all financial support and any other relevant financial or non-financial competing interests.<br><br>This statement is required for submission and **will appear in the published article** if	The authors have declared that no competing interests exist.

the submission is accepted. Please make sure it is accurate and that any funding sources listed in your Funding Information later in the submission form are also declared in your Financial Disclosure statement.<br><br>View published research articles from *PLOS ONE* for specific examples.<br><br>**NO authors have competing interests**<br><br>Enter: *The authors have declared that no competing interests exist.*<br><br>**Authors with competing interests**<br><br>Enter competing interest details beginning with this statement:<br><br>*I have read the journal's policy and the authors of this manuscript have the following competing interests: [insert competing interests here]*<br><br>* typeset	
**Ethics Statement**<br><br>Enter an ethics statement for this submission. This statement is required if the study involved:<br><br>• Human participants<br>• Human specimens or tissue<br>• Vertebrate animals or cephalopods<br>• Vertebrate embryos or tissues<br>• Field research<br><br>Write "N/A" if the submission does not require an ethics statement.<br><br>General guidance is provided below. Consult the submission guidelines for detailed instructions. **Make sure that all information entered here is included in the Methods section of the manuscript.**	The MUHAS Institutional Review Board granted ethical approval to conduct this study with approval number DA.282/298/01.C/2049. Permission to perform this study was also obtained from JKCI and Muhimbili National Hospital for laboratory investigations at the Central Pathology Laboratory (CPL). Consent to participate in this study was obtained from the patient by signing a written informed consent form. This study caused no harm to the study participants. For the participants who had positive UTI tests, results were communicated to the primary care physician for management, and all other participants' information was confidential

Powered by Editorial Manager® and ProduXion Manager® from Aries Systems Corporation

**Format for specific study types**<br><br>**Human Subject Research (involving human participants and/or tissue)**<br>• Give the name of the institutional review board or ethics committee that approved the study<br>• Include the approval number and/or a statement indicating approval of this research<br>• Indicate the form of consent obtained (written/oral) or the reason that consent was not obtained (e.g. the data were analyzed anonymously)<br><br>**Animal Research (involving vertebrate animals, embryos or tissues)**<br>• Provide the name of the Institutional Animal Care and Use Committee (IACUC) or other relevant ethics board that reviewed the study protocol, and indicate whether they approved this research or granted a formal waiver of ethical approval<br>• Include an approval number if one was obtained<br>• If the study involved *non-human primates*, add *additional details* about animal welfare and steps taken to ameliorate suffering<br>• If anesthesia, euthanasia, or any kind of animal sacrifice is part of the study, include briefly which substances and/or methods were applied<br><br>**Field Research**<br><br>Include the following details if this study involves the collection of plant, animal, or other materials from a natural setting:<br>• Field permit number<br>• Name of the institution or relevant body that granted permission	
**Data Availability**<br><br>Authors are required to make all data underlying the findings described fully available, without restriction, and from the time of publication. PLOS allows rare exceptions to address legal and ethical concerns. See the PLOS Data Policy and FAQ for detailed information.	No - some restrictions will apply

Powered by Editorial Manager® and ProduXion Manager® from Aries Systems Corporation

A Data Availability Statement describing where the data can be found is required at submission. Your answers to this question constitute the Data Availability Statement and **will be published in the article**, if accepted.<br><br>**Important:** Stating 'data available on request from the author' is not sufficient. If your data are only available upon request, select 'No' for the first question and explain your exceptional situation in the text box.<br><br>Do the authors confirm that all data underlying the findings described in their manuscript are fully available without restriction?	
**Describe where the data may be found in full sentences. If you are copying our sample text, replace any instances of XXX with the appropriate details.**<br><br>• If the data are **held or will be held in a public repository**, include URLs, accession numbers or DOIs. If this information will only be available after acceptance, indicate this by ticking the box below. For example: *All XXX files are available from the XXX database (accession number(s) XXX, XXX.).*<br>• If the data are all contained **within the manuscript and/or Supporting Information files**, enter the following: *All relevant data are within the manuscript and its Supporting Information files.*<br>• If neither of these applies but you are able to provide **details of access elsewhere**, with or without limitations, please do so. For example:<br><br>*Data cannot be shared publicly because of [XXX]. Data are available from the XXX Institutional Data Access / Ethics Committee (contact via XXX) for researchers who meet the criteria for access to confidential data.*<br><br>*The data underlying the results presented in the study are available from (include the name of the third party*	We are still analyzing the dataset for the second publication. We will make data available once this process is completed.

and contact information or URL).<br>• This text is appropriate if the data are owned by a third party and authors do not have permission to share the data.<br><br>* typeset	
Additional data availability information:	

Powered by Editorial Manager® and ProduXion Manager® from Aries Systems Corporation

Manuscript

Click here to access/download;Manuscript;JULIETHs Manuscript.pdf

Urinary Tract Infections and Drug Resistance Patterns in Heart Failure Patients on Sodium-Glucose Transporter 2 Inhibitors: Evidence from Jakaya Kikwete Cardiac Institute in Tanzania

→ The risk factors should also be included in the title.

Julieth Kaaya[1], Martine A. Manguzu[2], Deus Buma[3], Hamu Mlyuka[2], Samwel Rweyemamu[4], Albert Ntukula[5], Helfrid B. Ilomo[3], Elias Bukundi[6], Raphael Sangeda[7], Ritah Mutagonda[2*]

Affiliations

[1]Department of Pharmacy, Muhimbili Orthopedic Institute, P.O Box 65474, Dar es Salaam, Tanzania.

[2]Department of Clinical Pharmacy and Pharmacology, Muhimbili University of Health and Allied Sciences, P.O Box 65013, Dar es Salaam, Tanzania.

[3]Department of Pharmacy, Muhimbili National Hospital, P.O Box 65000, Dar es Salaam, Tanzania.

[4]Jakaya Kikwete Cardiac Institute, P.O Box 65141, Dar es Salaam, Tanzania.

[5] Department of Microbiology, Central Pathology Laboratory, Muhimbili National Hospital, P.O Box 65000, Dar es Salaam, Tanzania.

[6] Department of Epidemiology and Biostatistics, School of Public Health and Social Sciences, Muhimbili University of Health and Allied Sciences, Dar es Salaam, Tanzania.

[7]Department of Pharmaceutical Microbiology, School of Pharmacy, Muhimbili University of Health and Allied Sciences, P.O Box 65013, Dar es Salaam, Tanzania.

*Corresponding author: Email: rittdavisrida@yahoo.com; ritah.mutagonda@muhas.ac.tz

Abstract

Background: Sodium-glucose co-transporter 2 (SGLT2) inhibitors such as dapagliflozin and empagliflozin are increasingly used in heart failure (HF) management due to their cardiovascular benefits. However, glycosuria induced by SGLT2 inhibition may increase the risk of urinary tract infections (UTIs). Limited data exist on UTI burden among HF patients on SGLT2 inhibitors in low-resource settings.

Objective: This study aimed to determine the prevalence of UTIs, associated factors, and antimicrobial susceptibility patterns of uropathogens in HF patients receiving SGLT2 inhibitors at the Jakaya Kikwete Cardiac Institute (JKCI) in Tanzania.

Methods: A hospital-based cross-sectional study was conducted from March to June 2024 among HF patients aged ≥18 years on SGLT2 inhibitors. Data were collected using structured questionnaires and medical records. Midstream urine samples were cultured and subjected to antimicrobial susceptibility testing using the Kirby-Bauer disk diffusion method per CLSI 2024 guidelines. Descriptive statistics and modified Poisson regression were used for analysis.

Since the CLSI 2024 guidelines are broad, specify the part of CLSI 2024 that you used.

Results: Out of 138 participants among those cultured, the prevalence of UTI was 15.9%. The most common uropathogen was *Escherichia coli* (50%), followed by *Klebsiella pneumoniae* (13.6%), *Pseudomonas aeruginosa* (9.1%), and *Candida spp.* (9.1%). Significant risk factors for UTI included age >60 years (aPR 3.77; 95% CI: 1.42–10.01), female sex (aPR 2.92; 95% CI: 1.19–7.15), and SGLT2 inhibitor use ≥4 months (aPR 3.19; 95% CI: 1.70–5.96). High resistance was observed in bacterial isolates against ampicillin

(100%), tetracycline (69.2%), and ceftazidime (53.8%), whereas high susceptibility was noted against nitrofurantoin (84.6%) and meropenem (100%).

Conclusion: UTIs are common among HF patients on SGLT2 inhibitors, with *E. coli* as the predominant pathogen and a concerning resistance to commonly used antibiotics. These findings underscore the need for routine urine culture and sensitivity testing to guide appropriate therapy and promote antimicrobial stewardship, particularly in resource-constrained settings.

Keywords: SGLT2 inhibitors, Heart failure, Urinary tract infections, Uropathogens, Antimicrobial resistance, Antibiotic susceptibility

Background

Sodium-glucose co-transporter 2 (SGLT2) inhibitors, including dapagliflozin, empagliflozin, canagliflozin, and sotagliflozin, are a class of oral antihyperglycemic agents that lower blood glucose levels by inhibiting renal glucose reabsorption in the proximal tubule [1]. In addition to their glucose-lowering effects, SGLT2 inhibitors have demonstrated significant cardiovascular and renal benefits, including reductions in blood pressure, hospitalization for heart failure, and cardiovascular mortality [2]. These findings have led to their expanded use beyond diabetes, particularly in the management of heart failure with reduced ejection fraction (HFrEF), where they are now recommended as part of guideline-directed medical therapy [3].

Despite these therapeutic advantages, SGLT2 inhibitors have been associated with an increased risk of genitourinary tract infections. The mechanism is attributed to glucosuria, which creates a glucose-rich urinary environment conducive to microbial growth [1,4].

While the link between SGLT2 inhibitors and genital mycotic infections is well established, evidence regarding their association with urinary tract infections (UTIs) remains inconsistent. Some studies have reported a higher incidence of UTIs in patients using SGLT2 inhibitors, whereas others have found no significant difference compared to non-users. Reported cases of UTIs range in severity from uncomplicated cystitis to more serious infections such as pyelonephritis and urosepsis [3].

As the use of SGLT2 inhibitors continues to increase, particularly among patients with heart failure, it is important to evaluate their safety profile in different settings. In low- and middle-income countries, where factors such as limited access to hygiene and healthcare resources may further influence infection risk, understanding the burden of SGLT2 inhibitor-associated UTIs is critical. However, data on the prevalence of UTIs and antimicrobial resistance patterns in this population remain limited. This study was conducted to assess the prevalence of urinary tract infections, associated risk factors, and antimicrobial susceptibility patterns of uropathogens among heart failure patients receiving SGLT2 inhibitors at a tertiary cardiovascular institute in Tanzania.

Methods

Study design and setting

This hospital-based cross-sectional study was conducted at Jakaya Kikwete Cardiac Institute (JKCI), Dar es Salaam, Tanzania from 1st March to 30th June 2024. JKCI is a government-owned specialized and university teaching hospital offering cardiovascular care, training, and research services for the people of Tanzania and neighboring countries.

→ is this enough justification of the study site?

Study Population

The study population comprised heart failure patients aged 18 years with heart failure receiving an receiving SGLT2 inhibitors (i.e. either Empagliflozin or Dapagliflozin) and attended JKCI during the study period. Patients who were on antibiotics treatment within the last seven days or those with urethral catheters were excluded from the study.

Sample Size and Sampling Technique

Based on the available recent literature the prevalence of UTI among heart failure patients on SLGT2i is approximately 9.08%. A total of 141 participants were recruited consecutively based on a calculated sample size accounting for a 10% non-response rate. The sample size calculation formula for estimating a single proportion was used.

Data and specimen collection:

Using a structured questionnaire, data on socio-demographic characteristics, medical history, and medication details were recorded. A clean voided 10-20 milliliters of midstream urine specimens were collected in a wide screwed mouth container and transported within 10 minutes in a cool box to the Central Pathology Laboratory (CPL) at the microbiology department for culture and sensitivity testing.

Laboratory analysis: Culture, Isolation, and Identification of uropathogens

The urine sample was inoculated at the microbiology laboratory using a calibrated 1 microliter disposable loop in a cysteine, lactose electrolyte-deficient (CLED) agar, and blood agar media. After 24 hours of incubation at 35-37 °C, significant growth was further analyzed, and specific bacteria were isolated and identified through colony morphology,

gram reaction, and biochemical tests. A growth of $\geq 10^5$ Colony-forming units (CFU)/ml was required for statistical significance. Contamination was defined by the mixed growth of up to ≥104 more than two species. Bacterial isolation and identification were conducted using standard culture and biochemical tests. Urinalysis preceded urine culture on CLED and blood agar, with uropathogens identified by colony morphology and biochemical tests.

Antimicrobial Susceptibility Testing

Antimicrobial susceptibility testing (AST) was conducted using the Kirby Bauer disk diffusion method following CLSI guidelines (Clinical and Laboratory Standards Institute, 2024)(5).Bacterial suspensions were prepared by emulsifying one pure colony into a tube containing 2-5ml sterile normal saline (0.9% NaCl) and mixing gently until a homogeneous suspension formed. The suspension's turbidity was then adjusted to 0.5 McFarland standards to standardize the inoculum size. A sterile cotton swab was then dipped into the suspension and swabbed on the surface of the Mueller-Hinton agar plate. Standard antimicrobial-impregnated discs based on the uropathogens isolated were then aseptically placed on the culture.

The inoculated agar plates were incubated at 37 °C for 18-24 hours. The diameter of the zones of inhibition was measured in millimeters. The interpretation breakpoints were based on whether the bacteria were Susceptible (S), Intermediate (I), or resistant (R) to the tested antibiotic according to CLSI recommendations(5). Based on the type of isolated microorganism and CLSI recommendations, the antibiotics discs that were used with their respective concentrations include Amikacin 30µg (AK), Ampicillin 10 µg (AMP), Amoxy-clavulanate 20/10 µg (AMC), Cefepime 30 µg (FEP), Ceftazidime 30 µg (CAZ),

Ceftriaxone 30 µg (CRO), Cefoxitin 30 µg (FOX), Ciprofloxacin 5 µg (CIP), Gentamicin 10 µg (GE), Meropenem 10 µg (MEM), Nalidixic acid 30 µg (NA), Nitrofurantoin 300 µg (F), Penicillin G 10 units, Piperacillin-tazobactam 100/10 µg (TZP), Trimethoprim-sulfamethoxazole 1.25/23.75 µg (SXT), Tetracycline30 µg (TE), Tobramycin 10 µg (TM).

Data Analysis:

Data were analyzed in SPSS v26. Categorical variables were summarized as frequencies (%) and continuous variables as medians (IQR). Associations with UTI were evaluated using modified Poisson regression with robust variance, reporting crude (cPR) and adjusted prevalence ratios (aPR) with 95% CIs. Covariates were selected a priori and by bivariable screening ($p \leq 0.20$) with attention to model parsimony; a two-sided $p < 0.05$ denoted statistical significance.

Results

Social demographic and clinical characteristics

Most participants were males (55.3%) with a median age of ≤60 years. Most of the study participants (56%) were outpatients. The use of SGLT2 inhibitors was even between Empagliflozin 10mg (53.2%) and Dapagliflozin 10mg (46.8%). SGLT2 inhibitor use was predominantly less than 4 months (87%), **Table 1** and **Table 2.**

Table 1: Socio-demographic characteristics of the study participants

Variables	Frequency (n)	Percent (%)
**Age** (Years)		
≤60	76	53.9
>60	65	46.1
**Sex**		
Male	78	55.3
Female	63	44.7
**Menopausal status**		
Pre-menopausal	35	55.6
Post-menopausal	28	44.4
**Marital status**		
Married	99	70.2
Widow/Widower	26	18.4
Cohabiting	6	4.3
Single	5	3.5
Divorced	5	3.5
**Occupational status**		
Self-employed	79	56.0
Retired	37	26.2
Government/Private employed	22	15.6
Unemployed	2	1.4
Student	1	.7
**Bills coverage**		
Health insured	86	61.0
Cash	55	39.0
**Permanent Residence**		
Within Dar es Salaam	75	53.2
From other regions	61	43.3
From other countries	5	3.5
**Alcohol drinking habits**		
Yes	13	9.2
No	128	90.8

This categorization is too broad. It would help to break it down further.

This only applies to female why group it here?

Table 2: Clinical Characteristics and Medication History of the Study Participants

Variables	Frequency (n)	Percent (%)
**Patient admitted as**		
Outpatient	79	56.0
Inpatient	62	44.0
**Type of SGLT2 inhibitor used**		
Empagliflozin 10mg	75	53.2
Dapagliflozin 10mg	66	46.8
**Duration of SGLT2 inhibitor use**		
< 4 months	123	87
≥ 4 months	18	12.8
**Patient vitals**		
Median SBP (IQR) (mmHg)	112 (112, 134)	
Median DBP (IQR) (mmHg)	83 (78, 88)	
Median Pulse rate (IQR) (bpm)	88 (83, 92)	
**Left ventricular ejection fraction (LVEF) %**		
≤ 40 (HFrEF)	46	32.6
41-49 (HFmrEF)	58	41.1
≥ 50 (HFpEF)	37	26.2
**BMI status Kg/m^2**		
Underweight (≤18.5)	8	5.7
Normal weight (18.5-24.9)	57	40.4
Overweight (25.0-29.9)	44	31.2
Obese ≥30	32	22.7
**New York Heart Association Class (NYHA)**		
I&II	100	70.9
III	41	29.1
**Other Chronic Diseases**		
Yes	83	58.9
No	58	41.1
**Other Chronic Diseases**		
Other Cardiovascular diseases	53	63.9
Diabetes Mellitus	16	19.3
HIV/AIDS	3	3.6
Diabetes and other cardiovascular diseases	8	9.6
HIV/AIDS and other cardiovascular diseases	3	3.6
**Previous history of UTI**		
Yes	18	12.8
No	86	61.0
Unknown	36	26.2

Prevalence of UTIs and Risk factors → Present this data in an easier and better way.

138 samples were analyzed from 141 patients. Based on the culture and sensitivity results, the prevalence of UTIs among the participants was 15.9% Advanced age (>60 years) (adjusted prevalence ratio [aPR] 3.77, 95% CI: 1.42-10.01), female sex (aPR 2.92, 95% CI: 1.19-7.15), and longer duration of SGLT2 inhibitor use (≥4 months) (aPR 3.19, 95% CI: 1.70-5.96) were significantly associated with increased UTI risk.

Table 3: Univariable and multivariable analysis of the factors associated with urinary tract infection

		Univariable analysis			Multivariable analysis		
**Variable**	**Category**	**cPR**	**95% CI**	**p-value**	**aPR**	**95% CI**	**p-value**
**Age group**	≤ 60	Ref					
	>60	5.36	1.91 – 15.01	0.001	3.77	1.42 – 10.01	**0.008**
**Sex**	Male	Ref					
	Female	4.05	1.58 –10.35	0.004	2.92	1.19 – 7.15	**0.019**
**Admitted as**	Outpatient	1.11	0.51 – 2.43	0.791			
	Inpatient	Ref					
**SGLT2 inhibitor**	Empagliflozin 10mg	Ref					
	Dapagliflozin 10mg	0.66	0.30 -1.47	0.311			
**Duration of SGLT2i use**	< 4 months	Ref					
	≥ 4 Months	6.67	3.40 – 13.07	< 0.001	3.19	1.70 – 5.96	**< 0.001**
**Chronic diseases**	Yes	1.55	0.68 - 3.57	0.299			
	No	Ref					
**DM**	Yes	1.47	0.60 – 3.59	0.396			
	No	Ref					
**History of UTI**	Yes	3.78	1.74 – 8.23	**0.001**	1.58	0.90 – 2.79	0.113
	Unknown	0.97	0.33 – 2,89	0.958	0.85	0.37 – 1.99	0.713
	No	Ref					

Key: cPR: crude Prevalence Ratio, aPR: adjusted Prevalence Ratio, Ref: Reference, DM: Diabetes mellitus, NYHA: New York Heart Association, BMI: Body Mass Index

Uropathogens Identified

The predominant uropathogen was *Escherichia coli* (50%), followed by *Klebsiella pneumoniae* (13.6%), *Pseudomonas aeruginosa* (9.1%), *Candida spp* (9.1%), *Staphylococcus aureus* (4.5%), and *Staphylococcus saprophyticus* (4.5%) fig 1. – How did you ascertain their true identity? Gram negative and biochemical testing? → which ones did you use for candida and staphylococcus aureus and saprophyticus?

– Did you check the uropathogen isolation per age group? or why was it important to assess the sociodemographic characteristics of the patients?

– I recommend you show distribution of isolated pathogens in both outpatients and inpatients and their AMR profile.

– SGLT2 inhibitors too? is there a common trend for different SGLT2 used?

– Why was it important to check UTI history?

Antimicrobial Susceptibility Pattern

High resistance was observed against Ampicillin (99.9%), Tetracycline (69.2%), and Ceftazidime (53.8%). In contrast, uropathogens were highly susceptible to Nitrofurantoin (84.6%) and Meropenem (100%). Tables 4 and 5 show this.

Table 4: Antimicrobial resistance pattern of gram-negative bacteria isolated from urine culture

		**Antimicrobial agents resisted (%)**														
**Bacterial Isolates**	**n**	**AK**	**AMP**	**AMC**	**FEP**	**CAZ**	**CRO**	**CIP**	**GEN**	**MEM**	**NA**	**F**	**TZP**	**SXT**	**TE**	**TM**
Escherichia Coli	11	27.3	90.9	45.5	54.5	70.0	54.5	36.4	36.4	0	54.5	0	30.0	54.5	40.0	-
Klebsiella pneumoniae	3	33.3	100	33.3	33.3	33.3	33.3	33.3	33.3	0	33.3	33.3	0	66.7	66.7	-
Pseudomonas aeruginosa	2	100	-	-	0	100	-	0	-	50.0	-	-	100	-	-	0
Klebsiella oxytoca	2	0	100	50.0	0	0	0	0	50.0	0	0	50.0	0	50.0	0	-

Key: (-) Not applicable, AK: Amikacin, AMP: Ampicillin, AMC: Amoxicillin-Clavulanic acid, FEP: Cefepime, CAZ: Ceftazidime, CRO: Ceftriaxone, CIP: Ciprofloxacin, GE: Gentamycin, MEM: Meropenem, NA: Nalidixic acid, F: Nitrofurantoin, TZP: Piperacillin/tazobactam SXT: Trimethoprim/Sulfamethoxazole, TE: Tetracycline: Tobramycin. * why were some antimicrobials not tested?

Table 5: Antimicrobial resistance pattern of gram-positive bacteria isolated from urine culture

Revise this title.

		Antimicrobial agents resisted (%)						
**Bacterial Isolates**	**n**	**FOX**	**CIP**	**GE**	**F**	**Pen G**	**SXT**	**TE**
Staphylococcus aureus	1	0	100	0	0	0	0	100
Staphylococcus saprophyticus	1	-	0	100	0	100	100	100

Key:(-) Not applicable, FOX: Cefoxitin, CIP: Ciprofloxacin, GEN: Gentamycin, F: Nitrofurantoin, Pen G: Penicillin G, SXT: Sulfamethoxazole-trimethoprim, TE: Tetracycline.

15.6 or 15.9%. This needs to be constant.

Discussion

This study aimed to assess the prevalence of UTIs and antimicrobial susceptibility patterns of uropathogens isolated from heart failure patients on SGLT2 inhibitors. The study findings indicate a UTI prevalence of 15.6% in this patient population. This prevalence is higher compared to meta-analysis studies of the trials on the safety of SGLT2 inhibitors (4,6)A possible reason for the higher prevalence could have been the study site's water scarcity, which limits proper hygiene. Again, Tanzania is a tropical developing country with most of the summer season, and most warm weather leads to dehydration and decreased urine output, leading to an increased risk of UTI(7,8)All these studies have reported an increased risk, which is due to the mechanism of increased glucosuria, which creates an environment conducive to bacterial growth. (9).

Age older than 60 years was significantly associated with UTI, the most likely contributor to this risk is age-related changes in immune function and urinary tract anatomy and comorbidities(10). This is similarly reported in previous studies that investigated both diabetic and non-diabetic patients using SGLT2 inhibitors (11,12). Moreover, sex was also significantly associated with UTIs, female sex being at more risk; this finding aligns with known anatomical factors, such as the urethra being closer to the anus and the urethral opening being near the bladder, together with the hormonal factors that predispose women to UTI more than men (13).

How about the uropathogens and their AMR profiles? was it different in female?

Furthermore, the duration of SGLT2 inhibitors use was also a significant factor for UTIs among patients; those who had been on the medication for four months had a markedly higher risk of UTIs. This is due to persistent glucosuria, which increases the risk of bacteria proliferation and subsequent infection (14). This finding is like the study that studied the incidence of UTI among patients on SGLT2 inhibitors, which found that the occurrence of UTI was within three and six months of treatment (15). The observed late occurrence of UTI events of up to three months can

be explained by initial polyuria caused by the SGLT2 inhibitors; the diuresis acts to prevent the expected glucosuria, and the increased flow of urine through the tract decreases the urine bacterial load; therefore, preventing the proliferation of both bacteria and fungi (16)In this study, there was no difference in the risk of UTIs in patients taking either Dapagliflozin 10mg or Empagliflozin; however, there is a study that showed an increased risk of UTI with Dapagliflozin compared to Empagliflozin (17).

The study identified several uropathogens responsible for UTIs in this population, with Escherichia coli being the most frequently isolated pathogen (50%). This finding is consistent with global trends, where *E. coli* is the predominant uropathogen in both community-acquired and healthcare-associated UTIs (18). Other significant pathogens included *Klebsiella pneumoniae* (13%), *Pseudomonas aeruginosa* (9.1%), *Candida* spp. (9.1%), and *Klebsiella oxytoca* (9.1%). *Staphylococcus aureus* and *Staphylococcus saprophyticus* were responsible for 4.5% of the infections of Gram-positive organisms. The diversity of pathogens highlights the need for comprehensive diagnostic approaches to identify the causative organisms accurately and guide effective treatment.

Based on the isolated uropathogens, high resistance was noted that 100% and 90.9% of *Klebsiella* spp and *Escherichia coli* were resistant to Ampicillin like other studies in Tanzania (19,20), this persistent absolute resistance of Ampicillin even to other East African countries has led to the revocation of this product as a monotherapy in Tanzania by TMDA (21). The least resistance to no resistance was observed in Meropenem and Ciprofloxacin. *P. aeruginosa*, an alert micro-organism, has shown resistance to Amikacin, Piperacillin/tazobactam and is susceptible to Ciprofloxacin. The gram-positive bacteria are more resistant to tetracycline, *Staphylococcus saprophyticus* is resistant to gentamycin, penicillin G, and Sulfamethoxazole/trimethoprim that is susceptible to

Staphylococcus aureus, these gram-positive bacteria have shown a high susceptibility to nitrofurantoin which is also among the first-line treatment of UTIs. This resistance pattern is consistent with other studies (22–24)and shows the importance of culture and sensitivity testing to tailor antibiotic therapy effectively, thereby improving treatment outcomes and mitigating the development of antibiotic resistance. - Add more info about MDR and compare with other studies done.

Conclusion - The study concentrated more risk factors compared to uropathogen.
- The risk factors should then be captured in the title.
- I would also recommend adding the MDR data.

This study demonstrated that UTIs are prevalent among heart failure patients on SGLT2 inhibitors, with *Escherichia coli* being the most isolated pathogen. A notable resistance to commonly prescribed antibiotics indicates the need for culture-based diagnosis and sensitivity testing before initiating therapy. Furthermore, these findings suggest the need for an evidence-based decision in selecting empirical treatment for UTIs and thereby promoting the rational use of antibiotics.

This study further underscores the importance of UTI surveillance in HF patients on SGLT2 inhibitors, particularly in resource-limited settings. Targeted antimicrobial therapy and preventive measures are essential to improving patient outcomes and addressing AMR challenges in Tanzania.

List of Abbreviations

ADR Adverse Drug Reaction

AMR Antimicrobial Resistance

CFU Colony Forming Unit

CLSI Clinical and Laboratory Standards Institute

CPL Central Pathology Laboratory

HF Heart failure

HPF	High power field
JKCI	Jakaya Kikwete Cardiac Institute
NYHA	New York Heart Association
RCTs	Randomized Clinical Trials
SGLT2i	Sodium-glucose cotransporter 2 Inhibitor
TMDA	Tanzania Medicines and Medical Devices Authority
UPEC	Uropathogenic Escherichia coli
UTI	Urinary Tract Infections

Declaration

Ethical approval and consent to participate

The MUHAS Institutional Review Board granted ethical approval to conduct this study with approval number DA.282/298/01.C/2049. Permission to perform this study was also obtained from JKCI and Muhimbili National Hospital for laboratory investigations at the Central Pathology Laboratory (CPL). Consent to participate in this study was obtained from the patient by signing a written informed consent form. This study caused no harm to the study participants. For the participants who had positive UTI tests, results were communicated to the primary care physician for management, and all other participants' information was confidential.

Availability of data and materials

The data sets used and analyzed during the current study are available upon reasonable request from the corresponding author.

Competing interests

The author (s) declare that there are no competing interests.

Funding

This study was part of an academic qualification and did not receive any funds.

Acknowledgments

We would like to extend our gratitude to the Jakaya Kikwete Cardiac Institute (JKCI) for granting permission and providing support to conduct this study. Special thanks go to the microbiology staff members at CPL and other departments for their assistance in sample collection, culture, and testing procedures. We also appreciate the contributions of the Muhimbili University of Health and Allied Sciences (MUHAS) for their guidance throughout this research. Finally, we acknowledge the patients who participated in this study, without whom this research would not have been possible.

References

1. Fonseca-Correa JI, Correa-Rotter R. Sodium-Glucose Cotransporter 2 Inhibitors Mechanisms of Action: A Review. Vol. 8, Frontiers in Medicine. Frontiers Media S.A.; 2021.

2. Preda A, Montecucco F, Carbone F, Camici GG, Lüscher TF, Kraler S, et al. SGLT2 inhibitors: from glucose-lowering to cardiovascular benefits. Vol. 120, Cardiovascular Research. Oxford University Press; 2024. p. 443–60.

3. Vasilakou D, Karagiannis T, Athanasiadou E, Mainou M, Liakos A, Bekiari E, et al. Sodium–Glucose Cotransporter 2 Inhibitors for Type 2 Diabetes. Ann Intern Med. 2013 Aug 20;159(4):262–74.

4. Borovac JA, Kurir TT, Mustapic I, Kumric M, Bozic J, Glavas D, et al. SGLT2 inhibitors and the risk of urinary tract infections in patients with heart failure: A pooled analysis examining safety endpoints. Kardiol Pol. 2022;80(2):202–4.

5. James S. Lewis II PF. Performance Standards for Antimicrobial Susceptibility Testing, 34th Edition.

6. Deshpande R, Patel R, Regmi MR, Salih M, Kropp R, Al-Bast B, et al. Safety outcomes of sodium-glucose cotransporter-2 inhibitors in patients with type 2 diabetes and other risk factors for cardiovascular disease: a systematic review and meta-analysis. Cardiovasc Endocrinol Metab. 2023;12(2):1–10.

7. Simmering JE, Polgreen LA, Cavanaugh JE, Erickson BA, Suneja M, Polgreen PM. Warmer Weather and the Risk of Urinary Tract Infections in Women. Journal of Urology. 2021 Feb 1;205(2):500–6.

8. International Monentary Fund. UNITED REPUBLIC OF TANZANIA 12 INTERNATIONAL MONETARY FUND A. Stylized Facts of Climate Change Trends.

9. Islam MJ, Bagale K, John PP, Kurtz Z, Kulkarni R. Glycosuria Alters Uropathogenic Escherichia coli Global Gene Expression and Virulence. mSphere. 2022 Jun 29;7(3).

10. Storme O, Saucedo JT, Garcia-Mora A, Dehesa-Dávila M, Naber KG. Risk factors and predisposing conditions for urinary tract infection. Ther Adv Urol. 2019;11:19–28.

11. Khan S, Hashmi MS, Rana MA, Zafar GM, Asif S, Farooq MT, et al. Frequency of Urinary Tract Infections in Type 2 Diabetic Patients Taking Dapagliflozin. Cureus. 2022;14(1):7–11.

12. Yang H, Choi E, Park E, Na E, Chung SY, Kim B, et al. Risk of genital and urinary tract infections associated with SGLT-2 inhibitors as an add-on therapy to metformin in patients with type 2

diabetes mellitus: A retrospective cohort study in Korea. Vol. 10, Pharmacology Research and Perspectives. John Wiley and Sons Inc; 2022.

13. Czajkowski K, Broś-Konopielko M, Teliga-Czajkowska J. Urinary tract infection in women. Vol. 20, Przeglad Menopauzalny. Termedia Publishing House Ltd.; 2021. p. 40–7.

14. Dobrek L. Lower Urinary Tract Disorders as Adverse Drug Reactions—A Literature Review. Vol. 16, Pharmaceuticals. Multidisciplinary Digital Publishing Institute (MDPI); 2023.

15. Caro MKC, Cunanan EC, Kho SA. Incidence and factors associated with genitourinary infections among Type 2 diabetes patients on SGLT2 Inhibitors: A single retrospective cohort study. Diabetes Epidemiology and Management [Internet]. 2022;7:100082. Available from: https://doi.org/10.1016/j.deman.2022.100082

16. Milder TY, Stocker SL, Day RO, Greenfield JR. Potential Safety Issues with Use of Sodium-Glucose Cotransporter 2 Inhibitors, Particularly in People with Type 2 Diabetes and Chronic Kidney Disease. Drug Saf. 2020 Dec 1;43(12):1211–21.

17. Uitrakul S, Aksonnam K, Srivichai P, Wicheannarat S, Incomenoy S. The Incidence and Risk Factors of Urinary Tract Infection in Patients with Type 2 Diabetes Mellitus Using SGLT2 Inhibitors: A Real-World Observational Study. Medicines. 2022 Nov 22;9(12):59.

18. Flores-Mireles AL, Walker JN, Caparon M, Hultgren SJ. Urinary tract infections: Epidemiology, mechanisms of infection and treatment options. Vol. 13, Nature Reviews Microbiology. Nature Publishing Group; 2015. p. 269–84.

19. Mlugu EM, Mohamedi JA, Sangeda RZ, Mwambete KD. Prevalence of urinary tract infection and antimicrobial resistance patterns of uropathogens with biofilm forming capacity among outpatients in morogoro, Tanzania: a cross-sectional study. BMC Infect Dis. 2023;23(1):1–9.

20. Silago V, Moremi N, Mtebe M, Komba E, Masoud S, Mgaya FX, et al. Multidrug-Resistant Uropathogens Causing Community Acquired Urinary Tract Infections among Patients Attending Health Facilities in Mwanza and Dar es Salaam, Tanzania. Antibiotics (Basel) [Internet]. 2022 Nov 29;11(12). Available from: http://www.ncbi.nlm.nih.gov/pubmed/36551375

21. Tanzania Medicine and Medical Devices Authority. TMDA. Available from: https://www.tmda.go.tz/

22. Hrbacek J, Cermak P, Zachoval R. Current antibiotic resistance trends of uropathogens in central europe: Survey from a tertiary hospital urology department 2011–2019. Antibiotics. 2020;9(9):1–11.

23. Maldonado-barragán AA, Mshana SE, Keenan K, Ke X, Gillespie SH, Stelling J, et al. Predominance of multidrug-resistant (MDR) bacteria causing urinary tract infections (UTIs) among symptomatic patients in East Africa: a call for action. 2023;1–36. Available from: https://doi.org/10.1101/2023.06.13.23291274 ;

24. Kabugo D, Kizito S, Ashok DD, Graham KA, Nabimba R, Namunana S, et al. Factors associated with community-acquired urinary tract infections among adults attending assessment centre, Mulago Hospital Uganda. Afr Health Sci [Internet]. 2016 Dec;16(4):1131–42. Available from: http://www.ncbi.nlm.nih.gov/pubmed/28479906

List of figures

Figure 1: Proportion of isolated uropathogens among study participants' inhibitors with a positive culture

 Click here to access/download;Figure;Fig 1.pdf

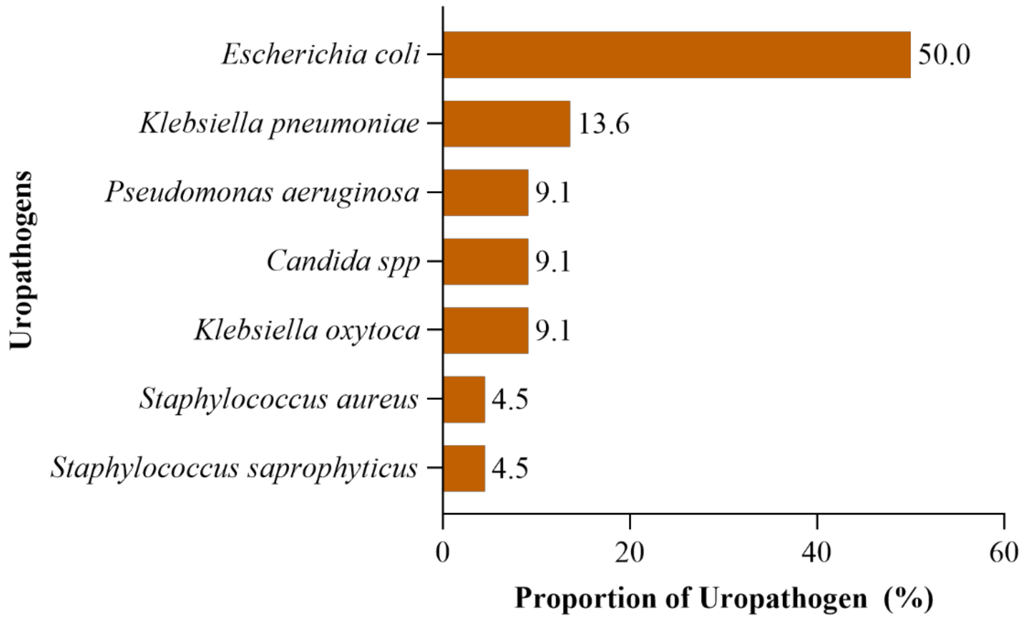

Supplement: Attachment — Submitted filename: JKCI study.pdf [file pone.0344986.s002.pdf]
