# Supplementary material for: Urinary tract infections, risk factors and antimicrobial resistance patterns in heart failure patients on sodium-glucose transporter 2 inhibitors: Evidence from Jakaya Kikwete Cardiac Institute in Tanzania
Source: PLoS One. 2026 Mar 26;21(3):e0344986. doi: 10.1371/journal.pone.0344986 (PMC13020780; doi:10.1371/journal.pone.0344986)
Supplement: Attachment — Submitted filename: Response to comment_PONE-D-25-48414.docx [file pone.0344986.s003.docx]

| **Reviewers’ Comment** | **Response** | **Revision Made** | **Page number** |
| --- | --- | --- | --- |
| **Reviewer 1** |  |  |  |
| *Title*  *“The risk factors should also be included in the title.”* | Thank you for this important suggestion. We agree that including the term *risk factors* in the title improves clarity and aligns with the study’s objectives. | The title has been revised to: **“Urinary Tract Infections, Risk Factors, and Antimicrobial Resistance Patterns among Heart Failure Patients on Sodium-Glucose Transporter 2 Inhibitors at Jakaya Kikwete Cardiac Institute in Tanzania.”** | Page 1, Line 1 |
| **Abstract** |  |  |  |
| *“Since the CLSI 2024 guidelines are broad, specify the part of CLSI 2024 that you used.”* | Thank you for this helpful clarification. We have now specified the exact CLSI guideline used for antimicrobial susceptibility interpretation to ensure methodological transparency. | The text in both the Abstract and Methods has been revised to state: “…interpreted according to CLSI M100 performance standards (CLSI, 2024).” (Abstract, Page 2; Methods Section, Page 5) | Abstract, page 2. Method section, page 6 |
| **Methods** |  |  |  |
| *“Is this enough justification for the study site?”* | Thank you for this important comment. We agree that further justification was necessary to clarify why JKCI was selected as the study site. | The description of the study setting was expanded to highlight that JKCI is the national cardiovascular referral center with a high volume of heart failure patients receiving SGLT2 inhibitors, making it an appropriate setting for this study. | Methods: Study Setting, Page 4) |
| *“Why this age group? Is there a scientific justification for this?”* | Thank you for this comment. We selected the age cut-off of ≥18 years because SGLT2 inhibitors are only approved and routinely prescribed for adults in heart failure management. | A justification has been added to the Study Population section explaining the ≥18 years age criterion | (Page 4, Study Population) |
| *“It would be better to show how you calculated the sample size.”* | Thank you for the suggestion. We have now provided the sample size calculation formula, parameters, and adjustment for non-response for clarity. | Sample size calculation formula and justification were added in the Sample Size and Sampling Technique section. | Page 5 |
| *“How did you ensure this was clean-void midstream?”* | Thank you for the observation. We added details on participant instruction and contamination control during specimen collection. | A statement on patient instruction and exclusion of contaminated samples has been added in the Data and Specimen Collection section. | Page 5 (data and specimen collection) |
| *“Which media did you use for colony enumeration?”* | Thank you for pointing this out. We clarified the specific roles of CLED and blood agar in microbial isolation. | Clarification added indicating that CLED agar was used for colony enumeration and blood agar for morphological identification. | Page 6 |

| **Reviewer Comment** | **Response** | **Revision Made** |
| --- | --- | --- |
| *“This section is too broad/repetitive. Also specify culture basis and contamination criteria.”* | Thank you for the helpful comment. We have now clarified the culture media roles, criteria for significant bacteriuria, and contamination thresholds. | Methods section updated to specify CLED for enumeration, blood agar for hemolysis, and contamination criteria. (Page 6) |
| *“Add more details about biochemical tests used for identification.”* | We agree with the reviewer. Additional details on the biochemical tests used to identify Gram-positive and Gram-negative isolates have been added. | Specific biochemical tests (catalase, coagulase, oxidase, indole, citrate) added to Laboratory Analysis. (Page 6) |
| *“Mention which antimicrobials were used in susceptibility testing.”* | Thank you. The antibiotics tested were already listed in the following paragraph. | AST section (Page 6 and 7) |
| *"Why did you not check multi-drug-resistant bacteria? It would have been interesting to see the MDR patterns."* | Thank you for this important observation. Unfortunately, MDR profiling was not performed because MDR classification criteria require testing antibiotic classes that were not uniformly available for all isolates during the study period. Therefore, performing MDR analysis would have introduced misclassification bias. However, we have added this as a **limitation** and recommended MDR analysis in future surveillance. | Limitation page 16 |
| **Results** |  |  |
| *"How did you ascertain SGLT2 usage?"* | Thank you for this comment. The method of verification for SGLT2 inhibitor exposure has now been clarified. Usage duration and dosage were confirmed using hospital electronic medical records and pharmacy dispensing logs. | Page 5, data and specimen collection |
| *“The age category appears broad*.” | Thank you for the suggestion. Age classification was maintained as ≤60 and >60 years to align with clinical literature indicating increased UTI risk associated with immune senescence in individuals above 60 years. |  |
| *Why include menopausal status since it only applies to women?* | Thank you for the observation. Menopausal status was included as it may influence susceptibility to UTIs due to estrogen decline. We have clarified that the variable was assessed only among female participants. |  |
| *“Present this data in an easier and better way.”* | Thank you for the constructive suggestion. We revised the paragraph on UTI prevalence and risk factors to improve clarity and readability and referenced Figure 1 for summarization. |  |
| *“How did you ascertain how these were identified? Which biochemical tests were used? What about Candida and Staphylococcus?”* | Thank you for this comment. We have expanded the Methods section to specify the Gram staining and biochemical tests used for bacterial identification and germ-tube testing for Candida spp. |  |
| *“Did you check uropathogen isolation per age group?”* | We appreciate the suggestion. Stratified analysis by age group was not performed because associations were examined using regression models, which already adjusted for age. This approach is statistically appropriate for estimating the independent effect of risk factors. | Page 10 |
| *“Show outpatient vs inpatient and SGLT2 inhibitor type distribution.”* | Thank you for this helpful comment. Stratifying uropathogen isolation by outpatient versus inpatient status and by SGLT2 inhibitor type was beyond the primary scope of the study and would have significantly reduced statistical power due to the small number of positive isolates. This limitation has now been acknowledged in the Study Limitations section. | Page 15 |
| *“Why was UTI history collected?”* | Thank you for this point. UTI history was collected to help differentiate recurrent infections and to assess predisposition to future episodes. |  |
| *“Why were some antimicrobials not tested?”* | Thank you for your observation. The antimicrobial susceptibility panel used in this study followed CLSI 2024 guidelines and the routine antibiotic testing practices at the study laboratory. Some antibiotics were not tested due to the unavailability of discs or because they are not included in the standard testing panel at our facility. |  |
| **Discussion** |  |  |
| *“15.6 or 15.9%? Must be consistent.”* | Thank you for this observation. We fixed the percentage to **15.9%** used throughout. | Page 13 |
| *“How about pathophysiology in females?”* | Thank you for this comment,  The explanation of the urethra length + vagina microbiota was explained in the second paragraph under sex as a risk factor | Page 13 |
| *“This needs to be compared with other studies.”* | Thank you for this comment, comparison added, but few studies are available on SGLT2 Inhibitors use, especially in Tanzania | Page 13 |
| *“This is a repetition of the results section.”* | Removed the restatement of percentages | Page 14 |
| *“Present in a better way.”, “Compare with other studies that also focus on UTI in patients on SGLT2 inhibitors in African and Developed countries.”,* | Flow improved | Page 14 |
| *“Add more info about MDR and compare with other studies done.”* | MDR analysis was not performed, and it is stated in the limitations | Page 15 |
| **Conclusion** |  |  |
| *“The study concentrated more on risk factors compared to uropathogens; the risk factors should then be captured in the title.”* | Revised the **title** to better reflect the dual focus on **UTI prevalence, risk factors, and antimicrobial susceptibility patterns** among heart failure patients on SGLT2 inhibitors. | Title |
| *“Should also recommend adding the MDR data.”* | Included in the **Study Limitations** that multi-drug resistant (MDR) organisms were not analyzed due to limited resources and sample size; recommended inclusion in future research. | Page 15, limitation section |
|  |  |  |
| **Reviewer 2** |  |  |
| “The title needs to capture risk factors.” (R#1) | We agree and revised the title to reflect the assessment of risk factors in addition to prevalence and antimicrobial resistance. Title updated to: *Urinary Tract Infections, Risk Factors, and Antimicrobial Resistance Patterns among Heart Failure Patients on SGLT2 Inhibitors: Evidence from Jakaya Kikwete Cardiac Institute, Tanzania.* | Page 1 |
| **Abstract** |  |  |
| Replace “midstream urine samples” with “midstream clean-catch urine samples.” (R#2) | Corrected throughout the abstract and manuscript for accuracy. | Page 2 |
| Use “semi-quantitative urine culture.” (R#2) | Correct terminology is adopted consistently. |  |
| “Out of 138 participants among those cultured” implies culturing patients. (R#2) | Revised to specify that **urine samples** were cultured, not patients. |  |
| Ensure all percentages carry one decimal place. (R#2) | Standardized to one decimal across the abstract. |  |
| Specify study population to emphasize applicability. (R#2) | Conclusion revised to explicitly refer to **HF patients on SGLT2 inhibitors**. |  |
| **Background** |  |  |
| Add comparative proportions/prevalence of UTI among HF patients on SGLT2i vs non-SGLT2i, including contradictory findings. (R#2 + orange) | Added paragraph summarizing comparative evidence; emphasized biologic plausibility via **glycosuria-facilitated colonization** (in line with our findings). | Page 3 |
| Clarify why the HF population matters. | Clarified HF-specific risks (e.g., comorbid diabetes, catheter exposure, reduced immunity) and clinical relevance of UTI outcomes in HF. |  |
| Improve transitions between epidemiology and rationale. | Rewrote transition sentences for coherence and flow. |  |
| **Methods** |  |  |
| “Why this age group (≥18 years)? Is there a scientific justification for this?” | We selected the age cut-off of ≥18 years because SGLT2 inhibitors are only approved and routinely prescribed for adults in heart failure management. | Page 4 |
| Do not italicize **“spp”/“species.”** (R#2) | Formatting corrected across Results and tables. | Throughout the document |
| **Results** |  |  |
| Begin characteristics with total number enrolled/analysed. (R#2) | Results now open with the total number of valid samples analysed. | Page 7. |
| Clarify why **138/141** were analysed. (R#2 + orange) | Explained that **3 samples were excluded due to contamination and insufficient volume** per rejection criteria. | Page 10 |
| State approach to >1 organism per sample. (R#2 + orange) | Mixed growth (≥2 organisms) was considered **contamination** and excluded; thus each positive culture contributed **one** isolation. |  |
| Provide actual counts with percentages (e.g., 50.0%; n=…). (R#2) | Tables/text now report **% and n** for each uropathogen. | Page 11. |
| Do not italicize **“spp”/“species.”** (R#2) | Formatting corrected across Results and tables. | Formatting revised. |
| One decimal place for all percentages. (R#2) | Standardized across Results/tables. | Formatting standardized. |
| Define **(TM)** and alphabetize abbreviations. (R#2) | Footnotes revised; abbreviations reordered alphabetically; **TM** defined. | Footnotes corrected. Page 12 |
| **Discussion** |  |  |
| Include prevalence estimates from meta-analyses; strengthen comparisons. (R#2) | Integrated relevant meta-analysis estimates comparing UTI risk in SGLT2i users vs non-users; positioned our findings within this context. | Page 13 and 14 |
| Correct spacing, missing full stops; improve flow. (R#2 | Comprehensive copy-editing performed to correct typography and enhance coherence. |  |
| “Clarify statement on late occurrence up to three months.” (R#2 | Clarified mechanism: initial osmotic diuresis may reduce adherence early; **persistent glycosuria** provides substrate favouring colonization later consistent with our observed timing. |  |
| Emphasize interpretation of key predictors (e.g., sex, diabetes, eGFR). | Strengthened interpretive paragraph linking clinical/biological plausibility to detected risk factors. |  |
| “Explain management implications for HF patients on SGLT2.” (R#2 | Added explicit recommendations: symptom-triggered **urinalysis + culture**, empirical therapy aligned to **local susceptibility**, **de-escalation** when AST available, and patient counselling on UTI symptoms. |  |
| **Study limitation and conclusion** |  |  |
| Add a distinct **Study Limitations** section before Conclusion. (R#2) | Inserted a concise limitations section (single-centre design; exclusion of contaminated samples may slightly underestimate prevalence; potential under-detection of asymptomatic bacteriuria; limited follow-up windows). | Page 15 |
| Specify the study population to improve applicability. (R#2) | Conclusion revised to explicitly reference **HF patients on SGLT2 inhibitors** and to recommend routine culture-guided therapy in symptomatic cases. | Page 15 |
|  |  |  |

**Editorial comments**

| **Section** | **Editor / Journal Requirement** | **Author Response** | **Revision Made (Page/Line – to insert)** |
| --- | --- | --- | --- |
| Table citation | “Ensure that **Table 3** is referred to in the text.” (Editor) | We inserted an explicit in-text reference to **Table 3** at the relevant Results paragraph. | Page 10 |
| Ethics placement | “Ethics statement should only appear in **Methods**.” (Editor) | Ethics statement was moved solely to the **Methods** section and removed elsewhere. | Page 5 |
| Data availability | Clarify open data plan (data to be freely accessible on acceptance). (Editor) | Data Availability Statement revised to comply with PLOS policy; dataset and codebook to be deposited in an open repository upon acceptance (accession link to be provided). | Statement updated. |
| Style & templates | PLOS ONE style and LaTeX template. (Editor) | Formatting revised according to PLOS style; LaTeX template notes acknowledged (content conforms to required structure and file naming). | Formatting checks completed. |
| Figures/pace tool | Upload figures to PACE (journal instruction). | Complied; figures prepared per PLOS specs, ready for PACE upload. | Noted in cover letter / submission checklist. |
| References | “Cite CLSI per suggested citation.” (R#2 + Editor note) | CLSI citation updated to the recommended **CLSI 2024** format; references checked for consistency. | Page 17 |
