# Supplementary material for: Urinary tract infections, risk factors and antimicrobial resistance patterns in heart failure patients on sodium-glucose transporter 2 inhibitors: Evidence from Jakaya Kikwete Cardiac Institute in Tanzania
Source: PLoS One. 2026 Mar 26;21(3):e0344986. doi: 10.1371/journal.pone.0344986 (PMC13020780; doi:10.1371/journal.pone.0344986)
Supplement: Attachment — Submitted filename: REVIEWER COMMENTS TABLE V2.docx [file pone.0344986.s004.docx]

**METHODS**

| **Section** | **Reviewer Comment** | **Author Response** | **Revision Made** |
| --- | --- | --- | --- |
| Methods – Laboratory Procedures | You should add more details on urinalysis, what criteria were used to determine if positive/negative. | The urinalysis procedure was expanded to clearly describe the method used (dipstick and/or microscopy) and the criteria applied to define positivity, including leukocyte esterase, nitrite positivity, and/or presence of pyuria. Although urinalysis findings were used as a screening tool, urine culture remained the reference standard for UTI diagnosis, and only culture-confirmed cases were included in the final analysis. | Additional description of urinalysis methodology and positivity criteria was inserted in the Methods section under Laboratory Procedures. (Page 6) |
| Methods – Antimicrobial Susceptibility Testing | Did both Gram-negative (GN) and Gram-positive (GP) bacteria have the same antimicrobial panels? | Antimicrobial discs were selected according to the type of isolated microorganism in line with CLSI M100 (2024) recommendations. Gram-negative and Gram-positive organisms were tested using organism-appropriate antimicrobial panels. | A clarifying sentence was added in the AST paragraph specifying that antimicrobial panels differed based on organism type. (Page 7) |
| Methods – Antimicrobial Susceptibility Testing | Why did you not check multidrug-resistant bacteria (MDR)? | We appreciate this important observation. Although antimicrobial susceptibility testing was performed for multiple antibiotic classes, multidrug resistance (MDR) was not predefined as a primary or secondary study outcome, and therefore a formal MDR classification using the standard definition (non-susceptibility to ≥1 agent in ≥3 antimicrobial categories) was not conducted during analysis. We agree that inclusion of MDR profiling would strengthen the clinical interpretation of resistance patterns and recommend that future studies incorporate standardized MDR definitions in the analytical framework. | A statement explaining the absence of MDR testing was added under the Study Limitations subsection. (Page 15) |
| Methods – Antimicrobial Susceptibility Testing | Delete the word “one” in “one pure colony,” which may be misleading for organisms with small colony size such as *Enterococcus* spp or *Streptococcus* spp. | The wording was revised to align with CLSI standards. | The phrase “one pure colony” was replaced with “3–5 well-isolated colonies” in the AST paragraph. (Page 6) |

**RESULTS**

| **Section** | **Reviewer Comment** | **Author Response** | **Revision Made** |
| --- | --- | --- | --- |
| Results – Uropathogens Identified | State the total number of uropathogens identified from the 138 urine cultures. Clarify whether any samples yielded 2 significant organisms (≥10⁵ CFU/ml), resulting in >138 isolates. | We clarified that a total of 22 uropathogens were identified from 138 urine cultures, corresponding to 22 positive samples. No mixed growth was observed, and each culture-positive specimen yielded a single significant organism (≥10⁵ CFU/ml). | Clarifying sentence added in Results – Uropathogens Identified subsection (Page 11) |
| Results – Uropathogen distribution by age | Did you check uropathogen isolation per age group? | We reviewed the dataset and clarified that no distinct clustering of specific uropathogens by age group was observed. | Statement added in Results after pathogen distribution description (Page 11). |
| Results – Inpatient vs Outpatient distribution | Show outpatient vs inpatient distribution of uropathogens. | We examined the dataset and clarified the distribution: Pseudomonas aeruginosa isolates were observed among outpatients, while Candida species were identified in both inpatient and outpatient settings, with no clear clustering by admission status. | Short clarifying sentence added in Results – Uropathogens subsection (Page 11 ). |
| Results – SGLT2 inhibitor type trend | Is there a common trend of uropathogens across different SGLT2 inhibitors? | We analyzed pathogen distribution by SGLT2 inhibitor type and found no clear trend between empagliflozin and dapagliflozin users. Escherichia coli remained the predominant isolate across both groups. | Statement added at end of Uropathogens Identified subsection (Page 11) |
| Results – Table 4 | Define “(TM)” and arrange abbreviations alphabetically. | Antimicrobial abbreviations were reviewed and corrected to align with CLSI standards. “TM” was corrected to TOB (Tobramycin) and “GE” corrected to GEN (Gentamicin). All abbreviations were arranged alphabetically in the footnotes. | Table 4 footnote revised; abbreviations corrected and reordered alphabetically. (Page 12) |

**DISCUSSION**

| **Reviewer Comment** | **Author Response** | **Revision Made (Location)** |
| --- | --- | --- |
| How about the uropathogens and AMR profile — was it different in females? | Sex-stratified analysis of uropathogen distribution and antimicrobial resistance (AMR) profiles was not performed due to the limited number of culture-positive cases (n=22), which reduced statistical power for subgroup comparisons. However, female sex was independently associated with increased UTI risk in multivariable analysis. We have acknowledged this as a limitation and suggested sex-specific analysis in future studies. | Discussion section revised to clarify lack of stratified pathogen/AMR analysis by sex and added statement in Study Limitations section. (Page 15) |
| Difference in UTI patterns among empagliflozin and dapagliflozin users in the results | Additional analysis showed no statistically significant difference in UTI prevalence between empagliflozin and dapagliflozin users. This has been clarified in the Results and briefly emphasized in the Discussion to avoid overinterpretation. | Results section clarified under risk factor analysis; Discussion paragraph updated to state no observed difference between SGLT2 inhibitor types. (Page 14) |
| The sentence on late occurrence and polyuria was unclear. Clarify whether polyuria decreases after three months and whether early glucosuria is insufficient for colonization. | The paragraph was revised for clarity. We clarified that SGLT2 inhibitors induce an initial osmotic diuresis (polyuria) during early therapy, which tends to stabilize over time as patients reach steady-state glycosuria and volume adaptation. Persistent glucosuria beyond the initial phase may provide a favorable environment for microbial colonization. The statement suggesting “temporary reduction of glucosuria” was corrected for accuracy. | Discussion paragraph on duration of SGLT2 inhibitor use revised for physiological accuracy and clarity. (Page 14) |
